# Alligator presence influences colony site selection of long-legged wading birds through large scale facilitative nest protector relationship

**DOI:** 10.1038/s41598-020-80185-5

**Published:** 2021-01-13

**Authors:** Wray Gabel, Peter Frederick, Jabi Zabala

**Affiliations:** Department of Wildlife Ecology and Conservation, University of Florida, Gainesville, FL 32611 USA

**Keywords:** Ecology, Community ecology, Wetlands ecology, Ecology, Community ecology, Wetlands ecology

## Abstract

Positive ecological relationships, such as facilitation, are an important force in community organization. The effects of facilitative relationships can be strong enough to cause changes in the distributions of species and in many cases have evolved as a response to predation pressure, however, very little is known about this potential trend in vertebrate facilitative relationships. Predation is an important selective pressure that may strongly influence breeding site selection by nesting birds. The American Alligator (*Alligator mississippiensis*) facilitates a safer nesting location for wading birds (Ciconiiformes and Pelecaniformes) by deterring mammalian nest predators from breeding sites. However, alligators do not occur throughout the breeding range of most wading birds, and it is unclear whether alligator presence affects colony site selection. We predicted that nesting wading birds change colony site preferences when alligators are not present to serve as nest protectors. Within the northern fringe of alligator distribution we compared colony characteristics in locations where alligator presence was either likely or unlikely while controlling for availability of habitat. Wading birds preferred islands that were farther from the mainland and farther from landmasses > 5 ha when alligator presence was unlikely compared to when alligators were likely. These findings indicate that wading birds are seeking nesting locations that are less accessible to mammalian predators when alligators are not present, and that this requirement is relaxed when alligators are present. This study illustrates how a landscape-scale difference between realized and fundamental niche can result from a facilitative relationship in vertebrates.

## Introduction

Ecologists have long recognized the role of predation and competition as primary species interactions that shape natural communities^1^. However, positive ecological interactions are increasingly seen as an important force in community organization^1–3^. Facilitation is one such positive ecological interaction that occurs when the presence of one species alters the environment in a way that increases the survival or reproduction of another species^3–6^. As defined by Bronstein (2009), facilitation can be mutualistic or commensal. For example, swollen-thorn acacia trees (*Acacia* sp.) and certain ants (*Pseudomyrmex* sp.) have a mutualistic facilitative relationship, where the ants provide protection from natural enemies and in turn benefit by a food reward^4^. Epiphytes may have a commensal facilitative relationship with their host plants, which are unaffected while the epiphyte benefits by gaining greater access to sunlight^7^. While facilitation is best described among plant species (Brooker et al. 2008), there are fewer examples within the animal kingdom^9–12^. In both plants and animals, the effects of facilitative relationships can be strong enough to influence the distributions of species^1,4,13^, and in many cases as an evolved response to predation pressure^1,5^, but evidence of that in animal populations is more scarce. Nonetheless, protection from predation is a common facilitative effect, which is predicted to be most common in communities where predation pressure has a strong effect on survival and reproduction, and thus, a stronger selective force^3^.

Nest predation is one of the biggest drivers of reproductive success in birds^14^, and protective nesting associations are a geographically widespread type of predation refuge often sought through facilitation by nesting birds^15^. These protective nesting associations occur when one species nests near a hazardous or annoying species that drives away predators of the first species simply by defending its own territory^15–17^. While examples of nest protector relationships described in the literature are limited to nesting birds, parallels to protective nesting associations exist in other taxa. Unpalatable or unattractive plants less susceptible to herbivory, for example, have been shown to provide protection for their relatively palatable and vulnerable neighbors^18–20^. Descriptive studies of protective nesting associations can be found amongst birds in a variety of taxa^15,17,21–27^ and are generally assumed to be commensal, although few researchers have investigated benefits to the protective associates^15,28^. These nest protector relationships often affect the reproductive success of the protected species locally (reviewed in Haemig 2001, Freestone 2006) and can influence small-scale decisions of nest site selection within a home range. However, whether these associations influence distribution and habitat use of species over larger scales has been seldom studied^29^.

Nest predation by mammalian mesopredators such as raccoons (*Procyon lotor*) and Virginia opossums (*Didelphis virginiana*) is a major factor in determining reproductive success of nesting long-legged wading birds (Ciconiiformes and Pelecaniformes, e.g. herons, egrets, ibises, storks and spoonbills; Frederick and Collopy 1989a). Access to breeding sites by only a few predator individuals can result in destruction of nest contents and colony-wide nest abandonment^17,30,31^. Although wading birds are generally colonial nesters, there is almost no group or individual nest protection behavior, and there is no effective behavioral defense against mammalian predation^17,31–35^. Wading birds seem to rely on passive defense strategies such as selecting inaccessible breeding sites that preclude the presence and activity of predators.

Wading birds nest in large colonies and employ collective decision-making when establishing new colony locations and returning to previously used colonies^36,37^. Colony site selection is based in part on an evaluation of the risks of nest predation or disturbance^38–41^. Wading birds prefer colony site characteristics that reduce nest predation such as islands^31,42^, which create a buffer against land predators^34^, or by associating with a nest protector. Wading birds have been noted to nest exclusively on islands in the middle of large bays^43–45^ rather than in shallower wetlands, and islands isolated from the mainland may have decreased predation risks^46–51^, and may be occupied by nesting birds more consistently^51^. Raccoon predation in colonies increases significantly as water depth decreases to the point that raccoons can walk rather than wade^17,31,49,52–55^. Nesting directly over water or on islands with a protective moat of water probably encourages the protective effect of water-based nest protectors, such as crocodilians, by forcing nest predators to swim to access the colony, which makes them highly vulnerable to predation^46, 53, 55–57^.

Indeed, a facultative mutualistic nest protector relationship is known to exist between long-legged wading birds and the American Alligator (*Alligator mississippiensis*). In this positive ecological association, alligators facilitate a safer nesting location for wading birds by deterring mammalian nest predators from wading bird colonies, and alligators receive substantial energy from food in the form of fallen nestlings^58,59^. Wading birds are also attracted to nesting sites with alligators present^17^. This mutualistic interaction between alligators and wading birds offers significant benefits for protector and protectee, despite being non-obligate, and illustrates how selective pressures of predation may have acted to form and reinforce a strongly positive ecological association. However, most of these apparent habitat preferences have been measured in the presence of alligators or other crocodilians, and it is unclear how they may be altered in the absence of alligators. Alligators do not occur throughout the entire breeding range of all species of wading birds in the United States, but mammalian predators do. It is unclear how the absence of alligators may alter the costs of reproduction for wading birds. Since colony site selection is the main known form of defense against mammalian predators, we predicted that colony site selection would be altered in the absence of alligators.

Here, we investigated whether the distribution of a nest protector can influence large-scale habitat use of protectees throughout their distribution area. We compare colony site preferences of nesting wading birds relative to protective characteristics of available colony sites and describe how those preferences change based on the probability of alligator presence/absence at the colony at the northern edge of the alligator’s present range. We predicted that in the absence of alligators wading birds would make increased use of islands, and that islands preferred by wading birds would be farther from features that attract or host land predators. In addition, we also predicted that wading birds would prefer colonies with environmental features that made access by raccoons harder or less enticing when alligator presence is unlikely, such as greater colony isolation from other colonies and from human development, lower percent composition of surrounding land with human development, taller nesting vegetation, and smaller sized islands. Alternatively, we predicted that when alligators were present at the colony wading birds would use them as nest protectors and their dependence on these alternative defense mechanisms would be relaxed. Both predictions are based on the understanding that islands and island distance from shore reduce accessibility to the colony by mammalian nest predators.

## Methods

### Study site

**Figure 1:**
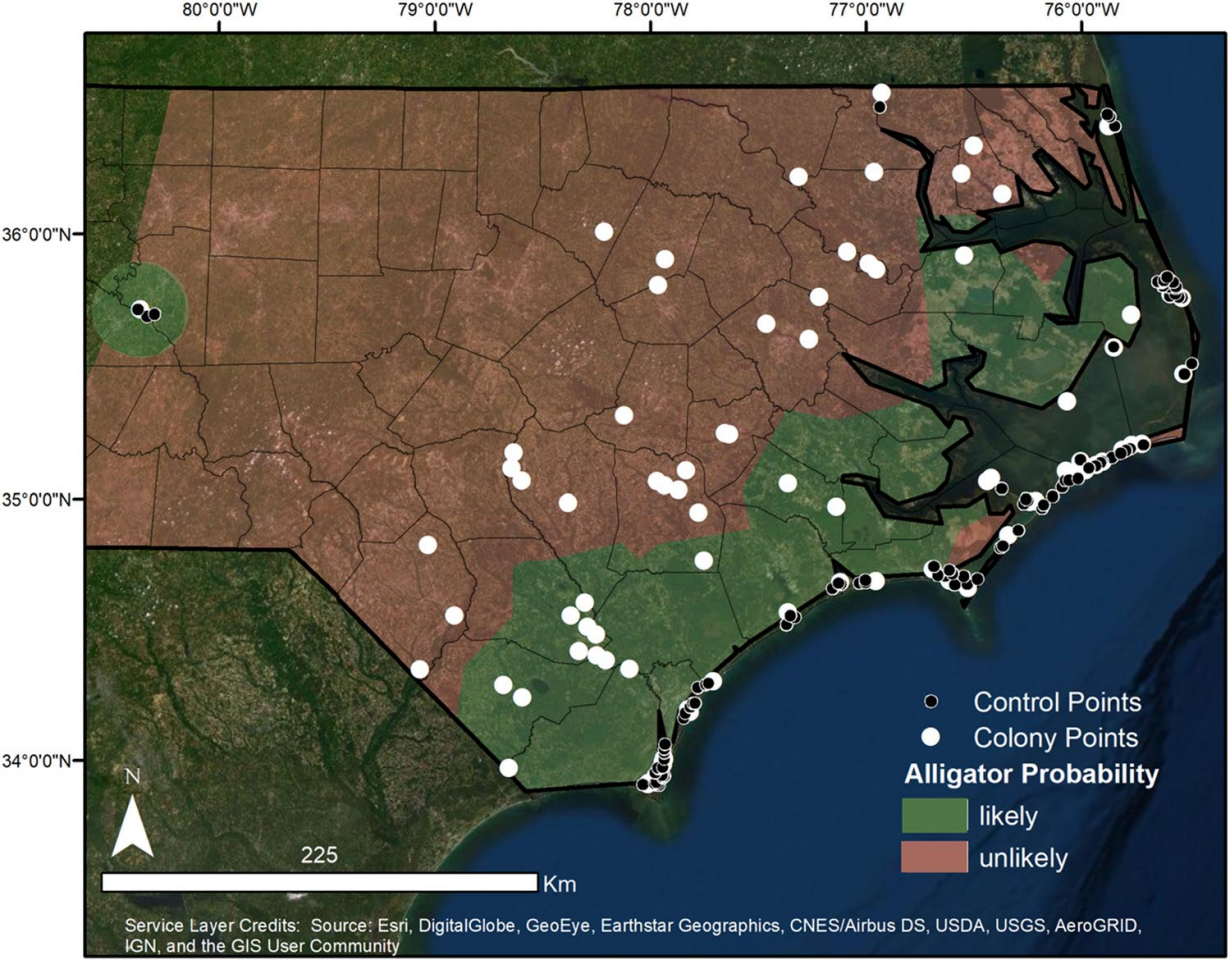
Map of the study area with locations for all wading bird colonies and control islands included in the analysis and the general alligator probability assignments throughout North Carolina. Solid white circles represent colony locations (island and mainland colonies) and small black circles represent available colony islands. Green blocks are areas where alligator occurrence is likely and red blocks are areas where alligator occurrence is unlikely. Note that available colony islands were only created for island colonies. Map generated in ESRI ArcMap 10.6^83^ (http://www.esri.com/). Main map satellite imagery is the World Imagery basemap within ArcGIS 10.6 software (http://www.esri.com/data/basemaps), see Service Layer Credits.

We studied wading bird colony locations in 28 counties in eastern North Carolina, predominantly in the Coastal Plain (57,565.8 km^2^): Wayne, Currituck, Gates, Nothampton, Perquimans, Dare, Franklin, Bertie, Nash, Martin, Washington, Davidson, Wilson, Pitt, Hyde, Lenoir, Sampson, Cumberland, Jones, Carteret, Duplin, Onslow, Robeson, Bladen, Pender, Columbus, New Hanover, and Brunswick (Fig. 1). The Coastal Plain is a geologically unified region that is flat, low lying, and includes rivers, marshes, and swamplands^60^. This area encompasses the northern extent of the alligator’s range^61–63^, and densities of alligators here are relatively low compared to more southern parts of their range^61,63,64^ as it encompasses areas where alligator presence is regular and others from which they are absent. This makes it ideal for comparing colonies with varying alligator occupancy probabilities, while avoiding variation resulting from geographic differences and survey methods. There is also extensive previous research describing current and historical alligator occupancy probabilities throughout this area^61,63,65^. Wading birds nest throughout the coastal plain in mixed species colonies^66–68^. Colonies were located on barrier islands, estuarine non-barrier islands, forested freshwater wetlands, impoundments, swamps/ponds, manmade/diked ponds, freshwater islands, and the shorelines of river streams. Colony sizes ranged from 3 to 2750 nests and colony substrates included dredged and diked materials, dredged and undiked materials, impoundments, and natural substrates.

Colonially nesting wading birds were surveyed periodically by the North Carolina Wildlife Resources Commission in coast-wide surveys^69^ and inland surveys (*Annual Performance Report* 1996), which vary slightly in methodologies that are described in detail in the Supplementary Methods S3 provided. Coastal surveys were conducted on foot following methods described by Soots Jr. and Parnell (1979) and Parnell and McCrimmon (1984), where 1–15 observers (depending on colony size) count active nests along a transect spaced 3–15 m apart. Inland surveys were conducted via fixed-wing aircraft at an altitude of 240 m and counts are confirmed with a ground survey. Colony species composition and numbers of nesting pairs of each species were recorded as well as colony site characteristics including percent vegetative cover, nesting vegetation height, and colony substrate.

We used colony locations in any year from 2000 to 2019. Colony composition consisted of Great Egrets (*Ardea alba*), Little Blue Herons (*Egretta caerulea*), Green Herons (*Butorides virescens*), Snowy Egrets (*Egretta thula*), and/or Tricolored Herons (*Egretta tricolor*), totaling 90 unique colonies (N = 90; Fig. 1). Of those 90 colonies, 44 were located on islands and 46 were located on mainland (Fig. 1). Wading bird colony locations were provided by the North Carolina Wildlife Resources Commission.

### Alligator presence probability

For each colony, we determined the probability that an alligator would be present at that site (classified as either likely or unlikely). Probability of alligator presence was determined using several sources of information about alligator population density and occurrence throughout North Carolina. These included assessments done by Gardner et al. (2016), Parlin et al. (2015), and O’Brien and Doerr (1986) and research grade iNaturalist observations^73^, and these determined probabilities were confirmed with general information about alligator physiological tolerances and limitations to ensure known tolerances of alligators matched our predictions^61,74–78^.

Alligator probability was judged to be likely (island = 24, mainland = 15) if the colony was located upstream or downstream ≤ 5 km of an area with a predicted occupancy probability > 40% (as calculated by Gardner et al. 2016), or it had ≥ 2 sensical iNaturalist sightings within 5 km (based on alligator core-use area calculated by Fujisaki et al. 2014) from the same year that the colony was observed. Colonies that did not meet those criteria had an alligator probability classified as unlikely (island = 20, mainland = 31). Previous research in North Carolina has shown that alligator occupancy and abundance decreases in more northern sites, in sites with higher salinity, and in sites that were generally more westward. Alligators in North Carolina are more likely to occur in coastal areas^61^ and typically don’t occupy barrier islands^63^. Although alligators do not prefer to continually reside in saline environments^80,81^, they will temporarily frequent marine influenced areas where salinities exceed those typically tolerated by alligators to forage^82^. Our alligator probability classifications match these observations and understandings of alligator environmental tolerances and behaviors (Fig. 1).

### Main methods of protection

Islands isolated from the mainland have decreased avian nest predation risks from terrestrial predators^46,48,49,51^, and wading birds seem to prefer nesting on islands because of this buffer^34,44,45,51^. We predicted that wading birds would prefer to nest on islands and that those islands would be farther from the mainland when alligator presence is unlikely. We identified islands using the North Carolina Center for Geographic Information and Analysis (CGIA) 1996 landcover vector digital data layer, which was produced through contract with Earth Satellite Corporation (EarthSat) in ESRI’s ArcGIS ArcMap software^83^. This layer had a 28.5 m resolution and 23 different land class classifications. Any land mass that was completely surrounded by open water (endpoint class 19) was defined as an island and this classification was confirmed using historically appropriate satellite imagery from the year the colony was surveyed. Any other landforms that were not islands were classified as mainland. We calculated the distance of each island colony to the nearest mainland.

We also considered the possibility that larger islands could sustain resident terrestrial mammalian predators. We predicted that wading birds would prefer to nest on islands that were farther from any landmass that could potentially host a raccoon when alligator presence is unlikely. Reported population densities of raccoons range from 1 raccoon every 5 ha to 1 raccoon every 43 ha^84–86^. According to this literature, islands with an area of less than 5 ha could not sustain a resident raccoon, so we also identified landmasses that were > 5 ha and grouped those with the mainland category, and then calculated the distance of each island colony to the nearest landmass that was > 5 ha.

### Alternative defensive strategies

At each colony site we also collected data on various other colony site characteristics that we hypothesized could be used by nesting wading birds as a defense against predators when alligator presence was unlikely. This included: colony distance from other colonies, colony distance to human development, percent composition of surrounding land with human development, vegetation height, and island size.

In addition to wading birds potentially isolating themselves from the mainland and from landmasses that could host raccoons, we thought wading birds might also seek out islands that are more isolated from other colonies when alligator presence is unlikely. We predicted that islands farther from other wading bird colonies would be less enticing to raccoons, who will readily travel between close colony islands^87^. We measured the shortest distance from the focal colony to the next nearest wading bird colony.

Raccoons are abundant and dense in human environments^88,89^, so we suspected that wading birds would avoid areas with human development when alligator presence is unlikely. We calculated the nearest distance from each colony to human development, as well as the percent composition of the land use type associated with human infrastructure within an 8.95 km buffer of the colony. We based the buffer distance on the average foraging distances from colonies that were reported for herons in eastern North America^90–95^. To determine the percent composition of human development for each colony we combined the low intensity development land class (endpoint class 2) and high intensity development land class (endpoint class 3) and calculated the total percent cover of the combined layer within the buffer. These categories included all areas where the land is covered predominantly by human structures, including densely populated urban and suburban areas^96^.

Within a colony, there are considerable differences in nesting site preference among species, but generally, the height of the nest above ground can be an effective method for deterring predators^47,97,98^. We predicted that the nesting vegetation used would be higher when alligator presence is unlikely. Vegetation height was a site-specific colony attribute that was categorized as low (0–99 cm) or high (1–7 m +) at the time of the wading bird colony survey.

Previous research has shown that island size is an important predictor of wading bird colony site selection^99^. Intermediate and small sized islands may be a better defense against mammalian predators than larger islands, which can potentially sustain a resident raccoon population or are otherwise more attractive to them^51,100^. For this reason, we hypothesized that wading birds would prefer smaller islands when alligator presence is unlikely. We measured the total area of each colony island.

### Generation of random points to account for availability

To better understand island-colony site preference relative to the availability of islands and possible variation within the study-site we used a use/availability design. We applied a buffer with an 8.95 km radius surrounding each island colony (see above for buffer distance justification), where resources and any other possible colony-sites within the buffer were deemed “available” and resources in the colony itself were deemed “used”. Within the buffer for each island colony we randomly generated 1–3 points (depending on availability) on other islands that represented plausible alternative colony locations that were considered available, yet unused (referred to as “available colony locations” hereafter; N = 102). Randomly generated points, thus, were on suitable unused islands that (1) were within the 8.95 km buffer of the related colony, (2) had a land class that was used by nesting wading birds within the study area, (3) had an island size ≥ the minimum observed colony island size (482 m^2^), (4) did not already host an active colony (or had not hosted one in the last 50 years based on historical data), and (5) did not already host another available colony location. Each available colony location had the same alligator probability classification as the associated active colony island. If there were more than three suitable available islands available for a given colony island then we selected three at random using ArcGIS Sampling Software^83^. We limited the maximum number of generated available colony locations per colony to ensure a balanced geographic representation of availability. To control for possible geographical variation in distances to mainland among colony and available islands from different regions we used the relative distance to mainland as a measure of distance rather than the actual distance. We calculated the relative colony distance to the mainland and to landmasses > 5 ha by subtracting the distance of the available colony island from the associated colony’s island distance. For example, a negative value indicated that the associated colony was comparatively closer to mainland than other available islands, while a positive value indicated that the associated colony island was farther from the mainland than the available island. For each available colony location, we generated the same data as the used colony location using the same methodologies (see above). We measured the available colony location’s distance to the nearest active colony, excluding the associated colony. We estimated vegetation height classifications at available points based on satellite imagery provided by Google Earth^101^ subjectively by eye. We also calculated the relative value of each continuous variable (relative island size, relative percent composition of human development, relative colony distance to other colonies) following the same procedure. To get an idea of the availability of suitable islands throughout the entire study area we recorded the total number of islands within each colony buffer that were unused by wading birds but that matched the above criteria. We only assigned available island colonies to island wading bird colonies. We did not assign available colony locations for mainland colonies because it was not feasible given the available information of the landscape to define discrete units of available space as we had done with available islands.

### Data analysis

We inspected correlations among continuous variables, but none had a Spearman’s correlation coefficient (r_s_) > 0.5. We used Tukey’s method^102^ to identify and remove 5 outliers ranging above or below the 1.5 Inter Quartile Range based on relative distance to the mainland (defined above, N = 97 used and unused/available colony location islands). We compared the proportion of island colonies and mainland colonies against the proportion of islands used and islands available, with alligator probability using a Pearson’s two-tailed Chi-squared test of equal proportions. We compared colony longevity (number of successive years the colony was active) and colony size (total number of nests) between island and mainland colonies using a two-way ANOVA. For all statistical analyses the alpha was set to 0.05 and all analyses were conducted in R 3.4.3 (Team 2018).

To determine associations between alligator occurrence and relative island distance from the mainland and relative island distance from areas > 5 ha we ran a linear mixed-effects model using the lmer function implemented in the “lme4” package^103^. We ran two models, one using relative distance (defined above) to mainland as the response variable and another using relative distance to mainland or islands > 5 ha as the response variable. We included alligator presence as predictor variable, and alternative methods of protection as covariates. We also assessed interactions between alligator occupancy and continuous covariates to assess possible variation in their relevance to wading birds in relation to alligator presence/absence. We included latitude and longitude as continuous covariates in the model to account for possible geographical trends. To further control for the possible influence of geographical variation in our results we compared raw distances between colonies and controls in areas where alligators were likely and unlikely. To control for the possible influence on results of a few far-away islands in areas where alligators were absent we truncated the data-set, deleting colony islands ≥ 2500 m from the mainland. Next, we checked that there were no differences in distances among categories (alligator likely/unlikely) and then re-assessed differences in relative distance to mainland in relation to alligator presence probability. For this analysis we used a generalized linear mixed-effects models (GLMM) with a logit linking function and binomial error type^104^. In this model, colony used vs. colony available was the response variable and the full interaction between relative island distance and alligator probability was the predictor variable.

To account for possible pseudo spatial correlation, both models included a site random effect (associated colony id for related available islands). In each case (used vs. available response, relative distance to mainland response, distance to islands > 5 ha response) we determined the best model using a manual backward stepwise selection process and used AICc to compare resulting competitive models. As our interest was on model comparison rather than parameter estimation, we did not use the restricted maximum likelihood estimator (REML). All continuous variables in the models were scaled.

## Results

### Main methods of protection

Overall, wading birds nested on a small portion (8.9%) of apparently suitable islands in eastern North Carolina, and nested more often on islands when alligator presence was likely than when unlikely (Pearson’s X^2^ = 3.5591, N = 90, p = 0.0358). However, there was no significant difference in the propensity of wading birds to nest on islands relative to alligator probability after we considered the availability of islands in each of these areas (Pearson’s X^2^ = 0.0903, N = 950, p = 0.7638). So wading birds were apparently more likely to nest on islands when alligator presence was likely than when alligator presence was unlikely because there were more islands available in areas where alligators were likely. Thus, we found no evidence to support the prediction that wading birds are nesting on islands more often when alligator presence is unlikely.

**Figure 2:**
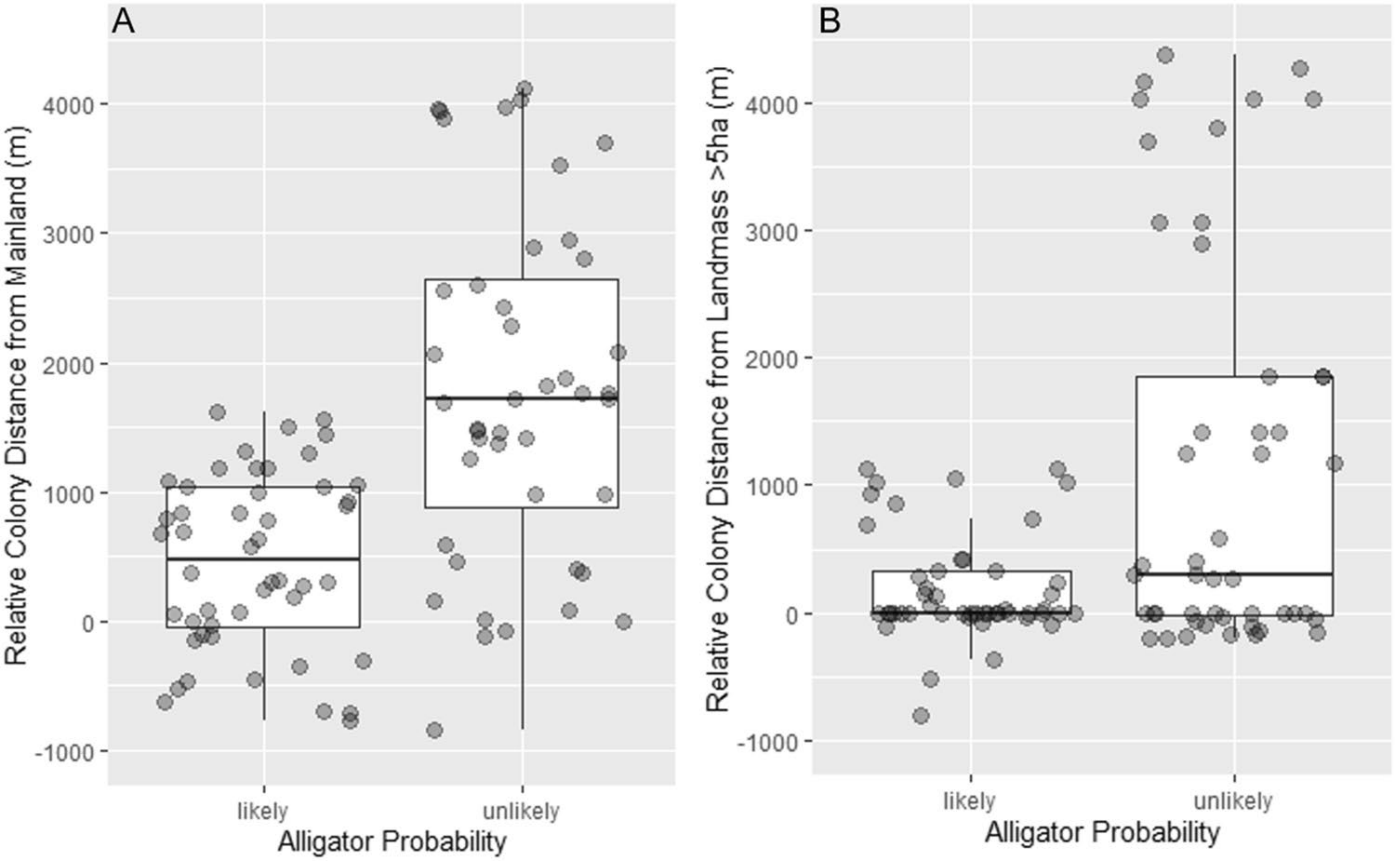
Colony island distance relative to control islands (meters) from (**A**) the mainland and from (**B**) landmasses > 5 ha, for areas with alligator probability of occurrence likely and unlikely. Please note that the distance represented in this figure is the relative distance of colony islands. Relative distance is the difference in distance between colony islands and control islands. For boxes, central line shows the median and boxes include all values within the 0.25 and 0.75 quantiles. Whiskers indicate range excluding outliers.

**Figure 3:**
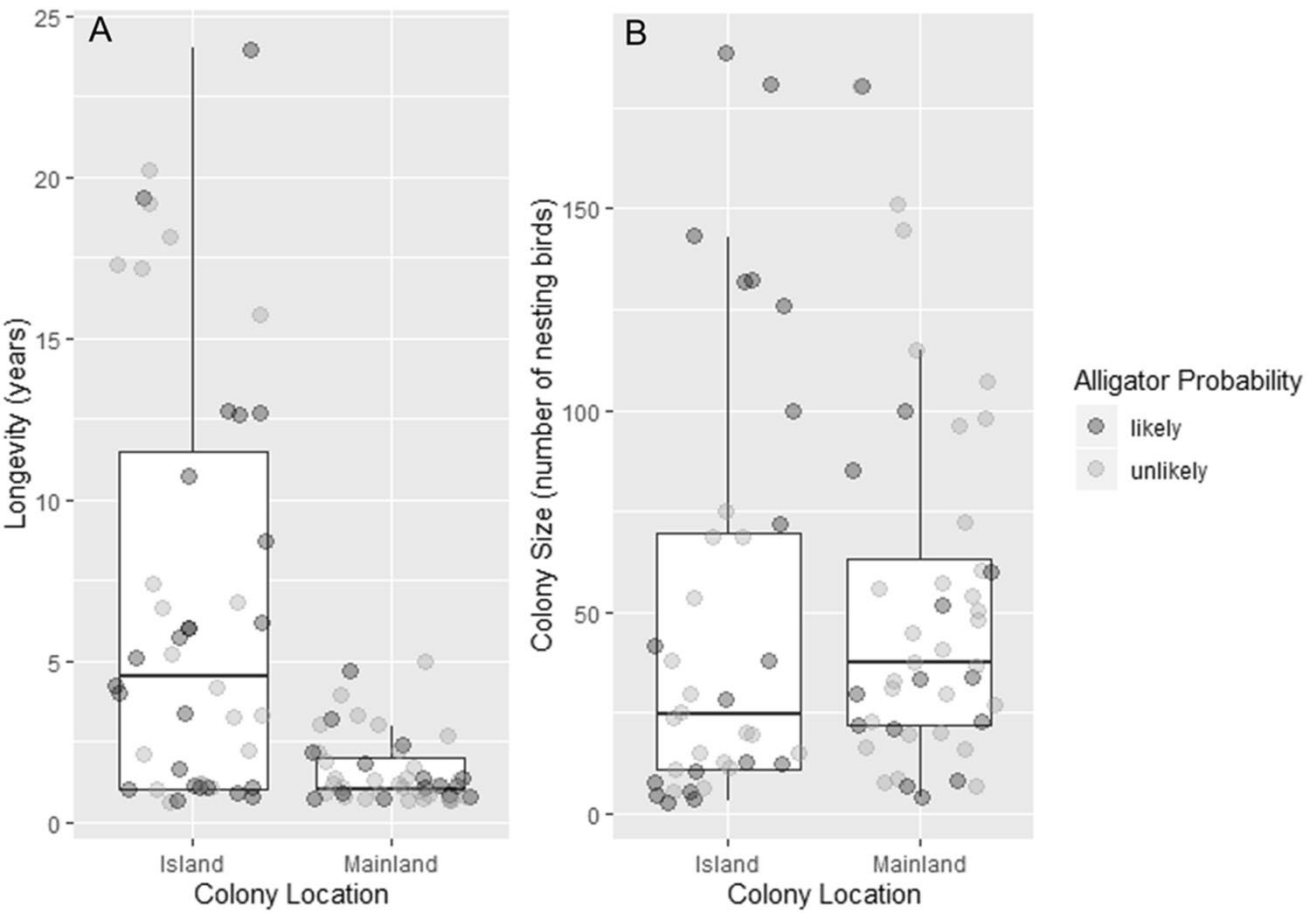
Mainland and island colony characteristics of (**A**) longevity (number of years) and (**B**) colony size (total number of nesting birds) relative to alligator probability. Likely alligator probability is represented by dark circles, unlikely alligator probability is represented by light circles. For boxes, central line shows the median and boxes include all values within the 0.25 and 0.75 quantiles. Whiskers indicate range excluding outliers.

However, colonies on islands where alligator presence was unlikely were farther from the mainland (Table 1A, Fig. 2A) and were also farther from any landmass > 5 ha (Table 1B, Fig. 2B), compared to available control islands. Colony island distance was highly spatially variable throughout the study area. In general, when alligator presence was unlikely, colony islands were an average of 913 m (± 1419, N = 50) from the mainland and an average of 254 m (± 795, N = 50) from landmasses > 5 ha. When alligator presence was likely, colony islands were an average of 720 m (± 767, N = 40) from the mainland and an average of 164 m (± 368, N = 40) from landmasses > 5 ha. To control for the possible influence of a few far-away colony islands in alligator-unlikely areas we truncated the data set by removing islands further than 2500 m from the mainland. In the resulting data set there were no differences in distances to mainland between islands in alligator likely/unlikely areas (β = − 166.71 ± 123.6, p = 0.179; see Supplementary Fig. S1), yet the differences in selected vs available islands, with birds in alligator unlikely areas selecting islands further away among these available remained significant (β = − 2.82 ± 0.87, p = 0.001; see Supplementary Fig. S2). Latitude and Longitude were not associated with relative island distance (latitude ΔAICc = 2.313, β = − 184.04 ± 231.17, p = 0.4313; longitude ΔAICc = 1.417, β = 208.29 ± 326.98, p = 0.2349) or the abundance of colony islands vs. available islands (latitude ΔAICc = 3.946, β = − 0.07 ± 0.27, p = 0.801; longitude ΔAICc = 3.946, β = 0.207 ± 0.277, p = 0.455). Colonies on the mainland (e.g. not on islands) had significantly lower longevity than colonies on islands (*F*(1,88) = 28.42, *p* < 0.0001; Fig. 3A), but did not have a significant difference in colony size (*F*(1,88) = 0.202, p = 0.654; Fig. 3B).

**Table 1:** Results of the best GLMM in each set assessing effect of alligator probability and alternative methods of protection on (A) relative colony distance from the mainland (meters) and (B) relative colony distance from landmasses > 5 ha (meters).

	Estimate	Standard error	t value	Pr( >|z|)
**(A) Relative colony distance from the mainland (m)**				
(Intercept)	380.46	200.81	1.895	0.0644
Alligator probability, unlikely	1234.14	276.03	4.471	< 0.001
Alligator probability, unlikely: distance to human development (m)	− 357.11	324.78	− 1.100	0.2744
Alligator probability, likely: distance to human development (m)	391.74	110.94	3.531	< 0.001
**(B) Relative colony distance from landmasses > 5 ha (m)**				
(Intercept)	257.22	249.74	1.030	0.3097
Alligator probability, unlikely	806.08	356.52	2.261	0.0298
Alligator probability, unlikely: distance to human development (m)	34.50	37.80	0.913	0.3651
Alligator probability, likely: distance to human development (m)	306.47	104.20	2.941	0.0047

Model includes associated colony as a random factor. All continuous variables were scaled.

### Alternative defense strategies

We did not find support for any association between distance from other colonies (ΔAICc = 2.32, β = − 141.57 ± 157.72, p = 0.3765), percent composition of human development within buffer (ΔAICc = 2.33, β = 6.51 ± 150.65, p = 0.9657), vegetation height (ΔAICc = 1.75, β = − 268.78 ± 188.17, p = 0.1574), or island size (ΔAICc = 2.15, β = − 24.74 ± 80.68, p = 0.7602) with alligator occupancy. We also found a significant correlation between an increase in relative distance of the colony from the mainland or from islands > 5 ha and an increase in distance to human development when alligators are unlikely (Table 1). However, that result seemed to arise from a partial correlation between distance to mainland and distance to human development areas in the residuals of the model as the correlation between raw variables was low (Spearman’s correlation coefficient = − 0.00376). Indeed, when we assessed the interaction between alligator occupancy and island distance, and independently included the interaction between alligator occupancy and distance to human development in the same model using island occupied or unoccupied as binomial response, the effect of human development on colony use was not significant within alligator occupancy likely (ΔAICc = 2.44, β = 0.26 ± 0.27, p = 0.339) or unlikely (ΔAICc = 2.44, β = 0.57 ± 0.59, p = 0.333) areas.

## Discussion

We hypothesized that wading birds were using alligators as nest protectors and that wading birds would change their colony site selection preferences based on alligator presence at the colony. When alligators were not available to serve as nest protectors, we predicted that wading birds would prefer sites that are farther from areas that may attract or host land predators. We found that wading birds actively select nesting islands that are farther from any landmass that could potentially contain mammalian predators when alligators are absent from colonies. Nest protector presence therefore appears to release wading birds from nesting site niche constraints associated with proximity to mainland, allowing them to nest in areas of the landscape that otherwise would be avoided.

Our results suggest that wading birds were no more or less likely to nest on islands vs mainland based on the presence of alligators. This may be because alligators are less mobile, and therefore, less successful at capturing prey within the colony in dry conditions or very shallow water, as is the case in mainland colonies, and so may be a poor deterrent for mammalian predators in these conditions^17,52,105,106^. Wading bird nests do experience greater predation by mammals in areas that are less inundated^31,49,52,55,107^, even in areas well within the range of alligators. Thus, this prediction might only be applicable to true islands in waters of a certain depth, and water may serve as a partial barrier and an effective deterrent to mammalian predators^34,108^ with or without alligators. Island colonies in this study also had greater longevity compared to mainland colonies (Fig. 3A), which is similar to a finding for Wood Storks (*Mycteria americana*) in the southeastern US^51^. We interpret this information to suggest that wading birds likely experience increased nest predation in mainland sites and may use them only where island sites are limited. Ultimately, water appears to be an effective deterrent for mammalian mesopredators regardless of alligator occupancy, and wading birds appear to always prefer nesting on islands over the mainland where colonies have decreased longevity. In addition to being an effective deterrent on its own, alligators are also better able to deter nest predators in colonies with water present due to improved mobility, which is consistent with our observations of wading bird colony site selection relative to alligator occupancy.

The idea that water itself may be an effective buffer to predation was further supported by wading bird preferences for islands that were farther from the mainland, and farther from other landmasses that could potentially host raccoons, than other available islands when alligator presence was unlikely. When alligator presence at the colony was unlikely wading birds selected islands to nest on that were, on average, 913 m from the mainland and when alligator presence at the colony was likely, wading birds selected islands that were, on average, 720 m from the mainland. However, it seems possible that even these distances may not completely eliminate mammals from colonies because many mammalian predators have substantial swimming abilities. North American raccoons readily make water crossings less than 400 m but have been observed swimming across open-water crossings of up to 950 m^109^. While this seems to indicate that colony distance from the mainland provides some protection from mammalian mesopredators, with the exception of the extreme end of the range of their swimming ability, it is unclear how island distance may affect tradeoffs (energetics or risk) for raccoons.

This work suggests that alligator occupancy is an important determinant of wading bird colony site selection preferences in North America. We would expect to see a continuation, and perhaps expansion, of the preference for islands that are farther from the mainland in more northern parts of wading bird range where wading birds are nesting well outside the farthest extent of alligators. Great Blue Herons (*Ardea herodias*) in Maine nested exclusively on islands even if it meant being farther from foraging areas^110^. On the other hand, we would expect to see wading birds continue to choose colony sites that are on islands closer to the mainland in more southern parts of wading bird range where alligators are more likely to be present. Colony locations in Florida, where alligators are ubiquitous, were not influenced by the distance to the mainland^111^, and wading birds in Louisiana, where alligators are ubiquitous too, preferred colony sites that were closer to the mainland over more distant and isolated islands^108^. Observed trends in wading bird colony site selection seen in northern areas outside the range of alligators, and in more southern sites in core areas of the alligator’s range, match trends expected from the extension of our results.

Facilitations include both tightly coevolved, mutually obligate associations as well as much looser facultative interactions^1^. Besides wading birds, alligators also beneficially affect many other organisms^112–119^, which range in strength of association. The American Alligator is considered a “keystone facilitator” because it modifies the local physical conditions in such a way that enables the existence of an entire community of other species^5^. The relationship between alligators and nesting wading birds is considered facultative because several species of wading birds do indeed nest outside the range of alligators, such as Great Egrets and Great Blue Herons. These larger sized wading birds may be better able to defend their nests from many kinds of mammalian predators and therefore may be more likely to engage in a nonobligate nest protector relationship with alligators than smaller sized herons, such as Little Blue Herons and Snowy Egrets. Smaller species of herons may have a more tightly evolved obligate facilitative relationship with alligators than their larger counterparts, which would be explained by their closely overlapping distributions in North America and supported by previous research describing a strong association between smaller *Egretta* herons and alligators^59^.

The evidence presented here supports the idea that facilitation can alter the relationship between the fundamental and realized niche as well as predictions of where a species can and will live in the physical world^1,6,120^. In this case, alligator occupancy apparently released additional wading bird nesting habitat by facilitating a greater number of colony sites safe from nest predators, and thus, allowing nesting wading birds to expand their realized niche in areas where these species distributions overlap. Specifically, in areas where alligator occupancy was likely, the presence of alligators allowed wading birds to occupy 35% more of available islands than if alligators had been predicted absent based on observed average colony distance in areas with and without alligators. This niche-based perspective on the effects of facilitation can provide us with a greater understanding of the role of nest protectors and other examples of animal-animal facilitation in community ecology at landscape scales.

This study contributes to a better understanding of the processes contributing to wading bird colonization of island habitats at a landscape level. We report large scale differences in habitat use associated with nest protector relationships. Nesting wading birds and crocodilians co-occur in many tropical and sub-tropical wetlands around the world (e.g., floodplains in Western Australia, the Amazon, India, Venezuelan Llanos, and Africa), and the relationship we describe here may exert similar influences over large parts of the world. Thus, our results potentially have wide geographical relevance. Additionally, our results have implications for climate change given that alligator distribution limits are most likely driven by cold temperatures^75^, and distributions of crocodilians may expand with a warming climate. This process could lead to an increase in available nesting habitat for nesting wading birds, and possibly, a range expansion for nesting wading birds.

Management projects and future studies of wading bird colonies within the range of alligators should include an in-person assessment of alligator occupancy to strengthen the validity of the results described here. There are also opportunities to better understand potential changes to wading bird preference of other environmental variables, including vegetation composition, relative to alligator occupancy.

## Supplementary information

Supplementary Information

Supplementary Information

Supplementary Information

## Data Availability

Our data contain sensitive biological information (site-specific information on occurrences of rare species) which restricts the distribution and use of such information to the public in an effort to protect species of concern, as well as to respect the rights of landowners.
