# Supplementary figures and images for: Alligator presence influences colony site selection of long-legged wading birds through large scale facilitative nest protector relationship
Source: Sci Rep. 2021 Jan 13;11:1019. doi: 10.1038/s41598-020-80185-5 (PMC7806806; doi:10.1038/s41598-020-80185-5)

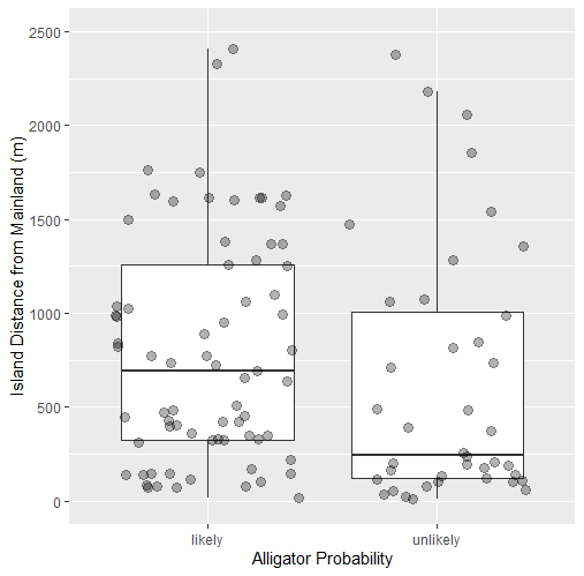

Supplement: Supplementary file 1 — Supplementary Information [file 41598_2020_80185_MOESM1_ESM.tif]

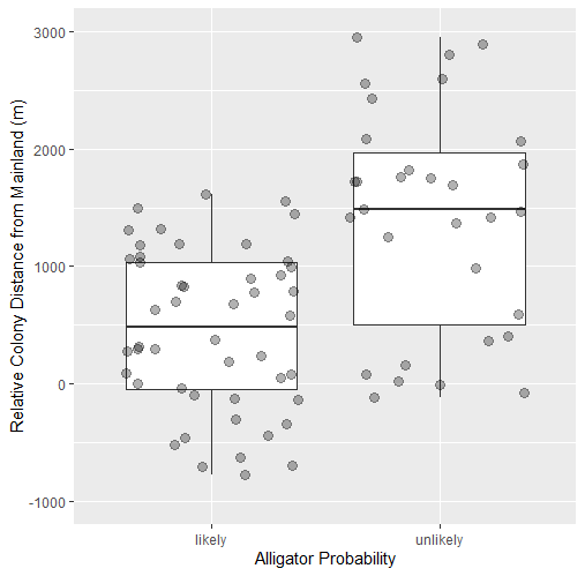

Supplement: Supplementary file 2 — Supplementary Information [file 41598_2020_80185_MOESM2_ESM.tif]
