# Supplementary material for: Alligator presence influences colony site selection of long-legged wading birds through large scale facilitative nest protector relationship
Source: Sci Rep. 2021 Jan 13;11:1019. doi: 10.1038/s41598-020-80185-5 (PMC7806806; doi:10.1038/s41598-020-80185-5)
Supplement: Supplementary file 3 — Supplementary Information [file 41598_2020_80185_MOESM3_ESM.docx]

Alligator presence influences colony site selection of long-legged wading birds through large scale facilitative nest protector relationship

Wray Gabel^1^, Peter Frederick^1*^, Jabi Zabala^1^

1) Department of Wildlife Ecology and Conservation. University of Florida, Gainesville, Florida 32611. U.S.A.

^*^ Corresponding author: [pfred@ufl.edu](mailto:pfred@ufl.edu)

ORCiD IDs: 0000-0002-7778-8823, 0000-0002-8098-9490, 0000-0002-1425-1594

Supplementary Methods: Wading bird colony survey method descriptions

The North Carolina Colonial Waterbird Database contains a history of all known nesting sites of colonial waterbirds in North Carolina. New colonies of wading birds are identified and surveyed using four different methodologies: coast-wide surveys, inland surveys, wood stork surveys, and occasionally opportunistically.

Coast-Wide Surveys: Conducted every three to four years (75, 76, 77, 83, 88, 93, 95, 97, 99, 01, 04, 07, 11, 14, 17) using methods described by Parnell and Soots (1979) and Parnell and McCrimmon (1984). Depending on colony size, 1-15 observers count active nests (defined as ≥1 egg or chick) along a transect spaced 3-15m apart. Complete ground counts are preferred, but if chicks are mobile colonies are then counted from the perimeter or the number of breeding pairs are estimated from adult counts.

Inland Surveys: Conducted less frequently (75, 76, 96, 08/09) than coast-wide surveys. All river basins, main river tributaries, and large swamps are surveyed from an altitude of 800’ by a fixed-winged aircraft. Once a colony is located it is circled, counted, and photographed and counts between multiple observers are averaged. A follow-up ground count is conducted for large colonies, colonies with a species of concern, and colony counts with a lot of uncertainty. Ground counts are done as close to aerial survey date as possible.

Wood Stork Surveys: Conducted annually since 2005 using fixed wing aircraft, UAV, kayak, and on foot. Periphery counts of Wood Stork nests are conducted from kayaks, counts from the ground and UAV are used to estimate numbers of active nests. Exact methods of survey vary slightly by colony. While this study did not include Wood Storks, these surveys often produced colony counts of other types of nesting wading birds that were of interest here.

Opportunistic Colony Finds: Any other surveys are from trusted people reporting species numbers from colonies that they encountered, either while conducting another survey, or some other activity. These trusted people often include land surveyors or botanists surveying in places that otherwise would not have been searched for wading bird colonies.

Supplementary Figures S1 and S2: Truncated island data (islands <2500m) results

Figure S1. Island distance (meters) from the mainland for areas with alligator probability of occurrence likely and unlikely based on a truncated dataset considering only islands <2500m from the mainland. For boxes, central line shows the median and boxes include all values within the 0.25 and 0.75 quantiles. Whiskers indicate range excluding outliers.

Figure S2. Colony island distance relative to control islands (meters) from the mainland for areas with alligator probability of occurrence likely and unlikely based on a truncated dataset considering only islands <2500m from the mainland. Please note that the distance represented in this figure is the relative distance of colony islands. Relative distance is the difference in distance between colony islands and control islands, for this reason there are some islands that have a relative distance >2500m. For boxes, central line shows the median and boxes include all values within the 0.25 and 0.75 quantiles. Whiskers indicate range excluding outliers.
